# Intellectual style theories: different types of categorizations and their relevance for practitioners

**DOI:** 10.1186/2193-1801-3-737

**Published:** 2014-12-15

**Authors:** Tine Nielsen

**Affiliations:** Department of Psychology, University of Copenhagen, Oester farimagsgade 2A, DK-1353 Copenhagen K, Denmark

**Keywords:** Choice of style theories, Types of categorizations of styles theories, Styles in practice

## Abstract

In the 20th century, a large number of psychological theories of intellectual styles were developed. Different reviews mention up to 71 theories of style. In the last 25 years, several suggestions as to how theories of styles may be divided into categories and fields of focus have been offered. Theorists and researchers disagree about the criteria on which categorizations should be based, and about which theories fulfill these criteria. Such disagreements are fruitful at a theoretical level, but also have negative consequences for the intended fields of application of the style theories and the associated instruments for measuring styles, because practitioners seeking the theory and instrument best suited for their intended use/application simply cannot find their way through the jungle of disagreements. The present study seeks to reduce the confusion for practitioners seeking to employ styles, by developing a taxonomy of categorizations of style theories in which all style theories can be placed.

**Electronic supplementary material:**

The online version of this article (doi:10.1186/2193-1801-3-737) contains supplementary material, which is available to authorized users.

## Introduction

During the second half of the 20th century, a large number of psychological theories of intellectual styles were developed. The term intellectual styles is an umbrella term proposed by Zhang and Sternberg (2005) to unify 10 theoretical frameworks of style, and it is gaining increasing use as an encompassing term for theories of cognitive, personality, learning, teaching and thinking styles, etc. In 1984 Messick (1984) stated that more than 19 cognitive styles could be defined at his time of writing. Seven years later, Riding and Cheema (1991) identified more than 30 different constructions of theories of styles designated cognitive styles or learning styles. In more recent reviews, the stated number of style theories is considerable higher, but also, in most cases, subject to a greater degree of uncertainty in labeling theories as theories of cognitive, learning, teaching, personality style, etc., and in some cases also a looseness in distinguishing theoretical models (or conceptual frameworks) from instruments. In an investigation of the applicability of learning style theories, Coffield et al. (2004) claimed to identify 71 theories of learning styles. In connection with Nielsen’s (2012) historical review of the styles literature, the count of what was referred to as style theories/conceptual models of styles, in 488 articles, reached 78, and 208 different instruments for the measurement of styles were referenced. However, the numbers can be expected to be somewhat, if not substantially larger, as the 488 articles reviewed in detail belonged to a larger pool of articles on styles (N = 2931). Evans and Waring's (2012) review of applications of styles in educational instruction and assessment identified 84 differently named style models.

Based on information such as the above, practitioners may be tempted to believe that a rapid development of style theories has been taking place, and indeed new theories (and instruments) have been developed through the years and are still being developed. However, the situation behind the number of theories in the different reviews is more a mixture of development of new theories, and the fact that the different reviews and summary works define both the overall concept of styles as well as the more specific style concepts in different ways. These differences in definitions are more often than not subtle – and as such elusive to the practitioner – but can have a big impact on the number of theories claimed to be theories of styles, as well as the number of theories claimed to specifically be theories of learning styles, cognitive styles, etc. For example, at the general level, at least one of Coffield et al.'s (2004) acclaimed theories of style was not as such a style theory, but rather a categorization model of style theories (i.e. Curry's onion model 1981). Another example at the general level, is the more recent article by Kozhevnikov et al. (2014), where the concepts of extrinsic-intrinsic motivation and tolerance for ambiguity were, quite unusually, claimed to be styles. An in depth look at the included reviews does, however, also show disagreement as to whether a number of other individual difference constructs are indeed style concepts or other types of individual differences, such as personality, ability and preferences for certain career fields or jobs (i.e. the Myers-Brigs personality types, Witkin's field-dependence/field-independence, Holland’s career-personality types).

Adding to the difficulties facing practitioners wanting to find a suitable theory of styles to apply in their own field of work are the many disagreements on the overall orientation or nature of the styles (for example cognitive or personality oriented styles) across the many different theories. Depending on the review or summary work consulted, a single theory may be categorized as a theory of cognitive styles in one review and a theory of personality styles in another review. Nielsen (2012) determined clearly that both theories and instruments were labeled somewhat arbitrarily as theories/instruments of learning, cognitive or teaching styles, in 488 articles reviewed in detail.

It is evident that the disagreements on the general as well as the more specific definitions of styles in the field have lead to many different categorizations of style theories based on different sets of criteria. Such disagreements are, of course, fruitful within the field of style theory and research as they stimulate further discussion, ideas and development. However, the same disagreements and the resulting different categorizations of theories of styles may very well have negative consequences for the intended fields of application of the theories and the associated instruments for measuring styles, because practitioners seeking the theory and instrument best suited for their intended use/application simply cannot find their way through this jungle of disagreements. Or worse still, the practitioners might not realize that it is a jungle, because as the concepts being used in reviews, which have sought to categorize and give an overview of the styles field, are the same word-wise, but defined quite differently, the resulting categorizations have appeared to be more alike than they really are.

The aim of the present study is to aid practitioners in finding some paths through this jungle and in choosing the "right" theory and measurement of style for their intended practical purpose. This will be achieved through a detailed analysis of a number of existing reviews and summary works, which categorize style theories, and placing these categorizations into a new proposed taxonomy for the organization (or categorization) of style theory categorizations, with three main types of categorization. This analysis will bring forward commonalities and differences between the different theory categorizations, thereby providing practitioners with useful information. That is, information on practice-related core concepts such as overall orientation of theories (cognitive, personality and/or learning/teaching styles), as well as stability and pedagogical amenability, which can aid the practitioner in his/her search for an appropriate theory within their intended area of use/application.

### The included reviews and summary works

The included reviews and summary works (Curry 1981; Messick 1984; Schmeck 1988; Jonassen and Grabowski 1993; Rayner and Riding 1997; Sternberg and Grigorenko 2001; Coffield et al. 2004; Zhang and Sternberg 2005) were, all but one, chosen as they are much cited and influential reviews or summary works in the styles field, when it comes to determining the specific nature of styles (i.e. categorizing style theories) as cognitive, learning, teaching, personality styles etc. The last included review (Kozhevnikov et al. 2014) was included as it represents the most recent attempt to categorize a number of style theories, and does this from quite another perspective than the previous works. In addition, the works all categorize style theories in different ways, which aided the purpose of developing the proposed taxonomy for categorizations of style theories. The included works cover a very long time range (i.e. 35 years), thereby also providing information on the development in the categorizations of styles theories over time.

### Proposed taxonomy of categorizations

#### Type A. Co-ordinate categorization of style theories with reference to sorting

Categories are defined as separate co-ordinate categories (there is no hierarchical relationship between the categories), so that a sorting of the theories of styles into these categories is achieved with the categorization.

#### Type B. Hierarchical categorization of style theories with reference to taxonomy

Categories are hierarchically constructed around one or more key characteristics of the theories, so that with the categorization, a taxonomy in relation to which theories of styles may be categorized is set up.

#### Type C. Hierarchical categorization of individual styles across theories with reference to taxonomy

Categories are hierarchically constructed around several key characteristics of individual styles, so that a sorting of individual styles across theories into a notional over-taxonomy is achieved with the categorization, so to speak.

## Type A co-ordinate categorizations with the classical disciplines of psychology as their point of departure

It is apparent that a number of the analyzed reviews (Jonassen and Grabowski 1993; Rayner and Riding 1997; Sternberg and Grigorenko 2001; Coffield et al. 2004) take the classical division of the disciplines of psychology as their point of departure. The categorizations of style theories in these reviews thus have in common that they all include two separate co-ordinate basic categories which reflect two of the classical disciplines of psychology, namely the cognition and personality-related disciplines. Furthermore, the four reviews have in common that they all (each in their own way) have found it necessary to supplement the cognitive and personality-related basic categories with one or more additional categories which cover different fields of psychology, but which do not constitute common classical psychological disciplines in themselves (Table 1).

**Table 1:** **Co-ordinate (type A) categorizations of theories of style with the classical disciplines of psychology as their common categories**

Common/shared categories of style theories as classical disciplines of psychology	Additional categories of style theories
	Learning oriented (Rayner and Riding 1997)
Cognition oriented	Activity oriented (Sternberg and Grigorenko 2001)
Personality oriented	Cognitive controls
	Learning styles
	(Jonassen and Grabowski 1993)
	Constitutionally based
	Flexible stable learning preferences
	Learning approaches, strategies, orientations and conceptions of learning (Coffield et al. 2004)

The first two reviews in Table 1 (Rayner and Riding 1997 and Sternberg and Grigorenko 2001) implement a pure type A categorization, i.e. they solely use the co-ordinate categories to sort the theories of styles. In both of these works, a single co-ordinate category is added to the two classical categories (cognition and personality oriented style theories), i.e. learning and activity oriented style theories respectively, where the latter in fact covers theories of learning and/or teaching styles. However, since these two reviews are concerned with different segments of the field of style theories, there is only slight concurrence in the theories placed in this third co-ordinate category; Kolb’s theory of experiential learning (Kolb 1984) and Dunn & Dunn’s learning styles (Dunn and Dunn 1993) are the only two theories, which are present in both Rayner and Riding's (1997) learning oriented category and in Sternberg and Grigorenko's (2001) activity oriented category.

The last two reviews in Table 1 (Jonassen and Grabowski 1993 and Coffield et al. 2004) implement a combination of the type A and type B categorizations, as they construct a taxonomy of theories of styles around the two basic co-ordinate categories (cognition orientated and personality orientated) each based on a different criterion - this we will return to in section on type B categorizations.

Looking at all four reviews in Table 1, only slight concurrence was found in the categorizations of style theories into the cognitive and personality style categories respectively, across the reviews. Thus Witkin’s perceptual styles (Witkin 1962) and Kagan’s conceptual tempo styles (Kagan 1966) were categorized as cognition oriented theories of styles, while Myers-Briggs’ theory of personality types (Myers and McCaulley 1985) was categorized as a personality oriented style theory in all four reviews.

### A summative suggestion for a co-ordinate categorisation

**Figure 1:**
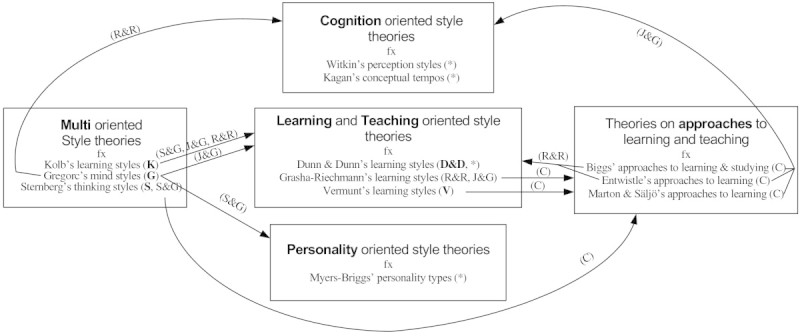
**A tentative categorization of a number of style theories into five co-ordinate categories.** Note. Brackets (…) indicate author initials for the reference works that places the particular style theory in the five categories respectively: J&G = Jonassen and Grabowski (1993), R&R = Rayner and Riding (1997), C = Coffield et al. (2004), S&G = Sternberg and Grigorenko (2001). b) (*) indicates agreement on the placing of a particular theory across the four reference works mentioned above. (bold author initials) indicate that the author through intensive reading of the particular theory found that this is the placing indicated by the theorist originator himself/herself: G = Gregorc (1982, 1998), S = Sternberg (1988, 1997), D&D = Dunn and Dunn (1993), K = Kolb (1984), Vermunt (1998).

Based on the agreements and differences in the categorizations in the four review works in Table 1, as well as a number of the theory originators’ own explanations of their theories, an attempt is made to unite all these similarities and discrepancies in categorizations in a new co-ordinate categorization (Figure 1). This new co-ordinate categorization contains five categories, which are mutually co-ordinate and not hierarchically or taxonomically related to one another. The vertical axis, inspired by Sternberg and Grigorenko (2001) and Rayner and Riding (1997), consists of the two classical categories; cognition and personality oriented theories of styles, with the category for learning and teaching oriented theories of styles placed in between these. The placement of the horizontal axis between the cognition and personality oriented styles theories is meant to imply that all of the theories on this axis draw on *both* of these classical psychological disciplines. The horizontal axis consists of the category learning and teaching oriented theories of styles in the middle, to which is added two categories: to the right, a category for theories on approaches to learning, which are style-like concepts, but strictly speaking not styles. To the left, a category of multi-oriented theories of styles, first suggested by Nielsen (2001). In Figure 1, brackets indicate where originators of a number of the theories as well as where the four reviews in Table 1 would place the given theories, and disagreements on the placement of a theory are illustrated by arrows pointing to alternative category placements according to other authors.

The placement of the individual theories of styles in the five categories in Figure 1 does not cover the field, rather it is an exemplification including only a selection of theories, which are recurring in the four review works in Table 1. Thus, only three of the classical theories in the cognition and personality oriented categories and two of the classical theories in the learning and teaching oriented category are included as examples in Figure 1. A number of the theories, which other categorizations place in these categories are placed in the so-called multi-oriented theories of styles, since these theories differ from the rest by operating with a higher-level concept of style (for example thinking styles or mind styles), which are in a sense more encompassing concepts than for example learning styles or teaching styles (Gregorc 1982, 1998; Sternberg 1988, 1997) These higher-level style concepts are a sort of umbrella (or meta) concepts as they can be construed as either work styles, learning styles, teaching styles, etc. dependent on the purpose and context they are employed in. They are however, only umbrella concepts in this sense, and not in the overall sense of the newer umbrella concept of intellectual styles, which has been proposed to encapsulate *all* style theories as well as theories on approaches to learning and teaching (Zhang and Sternberg 2005). Last, theories of approaches to learning and teaching are, in this model, included as an individual group of theories, since they define style-like concepts, but not styles in themselves (Schmeck 1988), even if Zhang & Sternberg’s inclusion of the approaches theories into the intellectual style umbrella concept (2005) appears to have been accepted without anybody in the field questioning this (Zhang et al. 2012).

The summative co-ordinate categorization of styles theories proposed in Figure 1 is considered useful to practitioners, because this type of categorization yields information as to the many different ways of categorizing styles theories proposed in various reviews and summary works, thereby giving the practitioner a brief overview of the confusion in the field as well as an opportunity to navigate through these disagreements according to their prevalent need for knowledge. For example, should a practitioner want to find a theory of learning and teaching styles to work with, one text might point out Gregorc’s mind styles (Gregorc 1982, 1998) as such a theory (Jonassen and Grabowski 1993), while other texts will place Gregorc’s theory of mind styles in a number of different categories of style theories: Sternberg and Grigorenko (2001) places Gregorc’s theory in the category of personality oriented style theories, Rayner and Riding (1997) places it in the category of cognition oriented style theories, and finally the author find that reading Gregorc’s own writings places the theory in the category of multi-oriented style theories. It is indeed no wonder that practitioners trying to choose a learning styles theory or a cognitive styles theory are having a hard time. Summative co-ordinate categorizations of styles theories, as proposed in Figure 1, are also useful to practitioners, because they demonstrate how the practitioner has to seeks out several sources of information to determine the nature of a particular style construct under consideration for practical use.

## Type B hierarchical categorization with reference to taxonomy – pedagogical susceptibility of learning styles

Two of the reviews (Curry 1981 & Coffield et al. 2004) analyzed employ what can be termed a type B categorization of style theories with reference to a taxonomy, as both of these works categorize style theories according to ordered criteria concerned with the stability of the styles in the different style theories. Styles are defined very differently within the different style theories, also with regard to their degree of (temporal) stability and the degree to which they are malleable. Whether particular styles are stable and/or malleable is crucial information to practitioners, as this determines the degree to which it is pedagogically possible to influence these styles, and the main purpose is often exactly to influence (i.e. change or develop) the thinking-wise preference and possibilities for learning in others. Zhang's (2013) extensive review shows that there is not sufficient empirical evidence as to the malleability of styles within individual theories, even if the existing research does support the notion of varying stability and of malleability of styles. Accordingly practitioners must rely primarily on the theoretical nature of the different styles, and secondarily on the empirical support, when searching for appropriate styles and instruments for practical application. Taxonomies such as Coffield et al.'s (2004) or Curry's (1981) are considered useful tools in this search, as they provide an overview of some style theories with regard to stability and/or malleability, from which the practitioner can narrow in their choice.

### Coffield et. al.’s families of learning styles theories

Inspired by Curry’s (1981) onion model, Coffield et al. (2004) present a categorization of 51 theories of styles in what they call *families of learning styles*. These families are seemingly defined based on how stable (and thereby susceptible to pedagogical influence) the learning styles in the theories in question are, and consequently, the categorization appears as a sort of stability taxonomy (Table 2).

**Table 2:** **Extract of Coffield et al.’s (** 2004 **) stability-taxonomy of learning styles theories**

Families of learning styles – description^a^	Example theories^b^
1. Learning styles and preferences are largely constitutionally based including the four modalities VAKT (visual, audio, kinesthetic, tactile)	Learning styles (Dunn and Dunn 1993)
	Mind styles (Gregorc 1982; 1988)
	Hemisphere dominance (Torrance in Torrance et al. 1976)
2. Learning styles reflect deep-seated features of the cognitive structure, including ’patterns of ability’.	Perception styles (Witkin 1962)
	Conceptual tempo (Kagan 1966)
	Intellectual structure (Guilford 1967)
3. Learning styles are one component of a relatively stable personality type	Personality types (Myers-Briggs in Myers and McCaulley 1985)
4. Learning styles are flexible stable learning preferences	Learning styles (Kolb 1984)
	Decision making styles (Kirton 1976)
5. Move on from learning styles to learning approaches, strategies, orientations and conceptions of learning	Approaches to learning (Entwistle 1988)
	Approaches to learning & studying (Biggs 1987)
	Approaches to learning (Marton & Säljö in Bowden and Marton 1998)
	Learning styles (Grasha and Riechmann 1975)
	Learning styles (Vermunt 1998)
	Thinking styles (Sternberg 1988, 1997)

Table notes. ^a^The descriptions are all cited from Coffield et al. (2004, p. 19). ^b^Only a selection of the theories included in Coffield et al. (2004) are included here.

The fundamental idea in Coffield et al.’s stability taxonomy (Table 2) is that the learning style theories placed in family 1 contain those learning styles which are the most stable and thereby also the least susceptible to pedagogical influence, while the learning styles in family 5 contain those learning styles which are the least stable and thereby also the most susceptible to pedagogical influence – the families in between these two have different degrees of stability. In Coffield et al.’s (2004) taxonomy, this ordering of the theories with regard to degree of stability has been determined by the extent of stability of individual models/theories claimed by the originators of the models. This approach is quite different from the one taken by Curry (1981), and it does yield a different taxonomy of stability. Whereas Curry places cognitive and personality oriented styles theories on the same level of stability, Coffield et al.’s approach results in family two (containing cognition oriented style theories) being viewed as relatively more stable than family three (containing personality oriented style theories) – an ordering which, depending on the particular theoretical position within these two psychological disciplines, will not always hold up.

Coffield et al.’s (2004) and Curry's stability taxonomies are also taxonomies for the possibility for pedagogically influencing or working with the learning styles in different ways, and as such they are helpful tools in the process of choosing a style theory for one’s own pedagogical practice. Thus, if a practitioner wishes to work pedagogically reflectively with matching of learning and teaching styles - for example by means of so-called pedagogical concordance and compensation strategies suggested by Nielsen (2006), which are sophisticated matching strategies including both matching and mismatching of teacher's styles and strategies to accommodate (and not) student's styles thereby also aiming at stylistic development, it would, according to Coffield et al.'s (2004) model, not be expedient to choose, for example, Dunn and Dunn’s (1993) theory of learning styles, since in this theory, the styles are regarded as stable and thereby unsusceptible to influence. Instead, according to Coffield et al.'s (2004) stability taxonomy, choosing for instance Kolb’s (1984) theory of experiential learning or Sternberg’s (1988, 1997) theory of mental self-government would be more appropriate, since in these theories, the styles are regarded as less stable and susceptible to different degrees of influence. If, instead, departure was taken in Curry's stability taxonomy, as presented in the original onion model from 1981, a somewhat opposite might result for a practitioner seeking a style theory for the purpose of affecting student's styles: Dunn and Dunn’s (1993) theory of learning styles are, in Curry's stability taxonomy, placed in the least stable and most pedagogically susceptible category; the instructional preferences in the outermost layer of the onion. Kolb's (1984) learning styles are placed in the middle layer, which contains the relatively less stable styles compared to the outer layer. And last, a theory as Witkin's (1962) perceptual styles is placed in the innermost layer of curry's model, which holds the rather stable styles. If, on the other hand, the practitioner should want to employ a more conventional and simple matching of teaching methods to students styles, as relayed in Paschler et al.'s (2008) critique of research related to the so-called matching hypothesis, where the aim is "effective learning", then the practitioner does not need to consider the stability or malleability of student styles.

## Type C hierarchical categorization across theories of styles with reference to taxonomy

As early as 1984, Messick stated that if an agreement of a core understanding of cognitive style could be reached within the field of theories of styles, it would be possible to lay down different types of style dimensions. Further, Messick discussed the attempts that had been made in that direction at his time of writing, and he arrived at 11 core understandings (or types) of cognitive style. These were, however, not mutually exclusive, which is why Messick did not arrive at a final categorization. Yet, the idea of a categorization of styles across theories has been re-addressed by Zhang and Sternberg (2005), who put forward a unifying threefold model of intellectual styles, and again most recently by Kozhevnikov et al. (2014) in their cognitive-style matrix.

### Zhang and Sternberg's threefold model of intellectual styles

Zhang and Sternberg’s (2005) threefold model of intellectual styles constitutes what is referred to in this paper as a type C categorization of theories of styles, i.e. a hierarchical categorization of individual styles across theories of styles with regard to several characteristics, so that an overall taxonomy of intellectual styles is achieved. Zhang and Sternberg’s characterization differs from the categorizations treated previously due to two factors. *First*, the categorization runs across theories and thus categorizes individual styles into three main types. *Second*, it is a case of a rather complicated taxonomy, since the placement of the individual styles into the three main types or main categories is determined by six different characteristics. These six characteristics are obviously inspired by other theorists’ categorization of theories of styles and by the actual descriptions of styles found in the individual theories (Messick 1984; Curry 1981; Coffield et al. 2004; Sternberg 1997; Kolb 1984 and Gregorc 1998, cf. Table 3).

**Table 3:** **Zhang and Sternberg’s (** 2005 **) threefold model of intellectual styles represented as a taxonomy of styles across style theories (type C categorization)** ^**a**^

The three style types ….			
Degree of structuring (structure – free of structure)	Low	High	varied^c^
Degree of cognitive complexity (simple – complex)	High	Low	varied^c^
Degree of conformity (nonconform – conform)	Low	High	varied^c^
Degree of management/control (autonomy – authority)	Low	High	Varied^c^
Style construct^b^	Type I styles	Type II styles	Type III styles
a. Learning approach	Deep	Surface	Achieving
b. Career-personality type	Artistic	Conventional	Realistic, Investigative, Social, Enterprising
c. Mode of thinking	Holistic	Analytic	Integrative
d. Personality type	Intuitive, Perceiving	Sensing, Judging	Thinking, Feeling, Introversion, Extraversion
e. Mind style	Concrete random	Concrete sequential	Abstract random, Abstract sequential
f. Decision-making style	Innovation	Adaptation	
g. Conceptual tempo	Reflectivity	Impulsivity	
h. Structure of intellect	Divergent thinking	Convergent thinking	
i. Perceptual style	Field-independence	Field-dependence	
j. Thinking style	Legislative, Judicial, Hierarchic, Global	Executive, Monarchic, Local, Conservative	Oligarchic, Anarchic, Democratic, Internal, external
The mutual relative value-ladenness of the three types of styles	High (positive)	High (negative)	Low-high (differentiated)
The mutual relative stability of the three types of styles	More	More	Less

Table notes. a) The table is the author’s combination of Table V and the different textual descriptions of the threefold model of intellectual styles in Zhang and Sternberg (2005). b) The theoretical background for the individual styles categorized in the three major types of styles are: *“*^*a*^*Bigs’s theory of student learning,*^*b*^*Holland’s theory of career-personality types,*^*c*^*Torrance’s construct of brain dominance,*^*d*^*Jung’s theory of personality types,*^*e*^*Gregorc’s model of mind styles,*^*f*^*Kirton’s model of decision-making styles,*^*g*^*Kagan’s model of reflectivity-impulsivity conceptual tempo,*^*h*^*Guilford’s model of structure of intellect,*^*i*^*Witkin’s construct of field-dependence/independence,*^*j*^*Sternberg’s theory of mental self-government”* (Zhang and Sternberg 2005, p. 35). The Democratic style proposed by Nielsen et al. (2007), as an extension of the theory of mental self-government, is considered to be a type III style, and as such included here for completeness. c) Dependent on the stylistic demands of a particular task and the individual’s interest in the task, these styles will manifest different degrees of preference on the four preference continua (top of table) as if they were in fact type I or type II styles.

The six characteristics used to categorize individual styles across ten theories may be divided into two different types. The first type consists of four different continua of preference which indicate how high an “absolute” degree of preference for the four characteristics mentioned below is connected to the individual style:

- Structuring (moving from unstructured to structured)
- Cognitive complexity (moving from simple to complex)
- Conformity (moving from nonconforming to conforming)
- Management/control (moving from autonomy to authority)

The second type of characteristics consists of the two more general continua of degree of value-ladenness and stability. However, with these characteristics, the degree to which they apply to the individual styles in the model is “relative” among the three types (I, II and III) of intellectual styles:

- The mutual relative value-ladenness of the three types of styles (from low to high, either positive or negative), and
- the mutual relative stability of the three types of styles (from low to high).

In this way Zhang and Sternberg (2005) places the individual styles in ten different theories of styles into the frame of the above six continua of characteristics so that the result is a taxonomy of three different types (I, II and III) of intellectual styles across the ten theories:

#### Type I intellectual styles

I.e. a preference for the structured, the cognitively complex, the nonconforming, and for autonomy. Relative to the two other types of intellectual styles, this type of styles is laden with relatively positive value, and compared to the third type of intellectual styles; it is relatively stable (Zhang and Sternberg 2005). Collectively, styles placed in this main category may be referred to as *the creative styles*.

#### Type II intellectual styles

I.e. a preference for the structured, the cognitively simple, the conforming, and for the authoritative. Relative to the two other types of intellectual styles, this type of styles is laden with inordinately negative value, and compared to the third type of intellectual styles; it is relatively stable (Zhang and Sternberg 2005). Collectively, styles placed in this main category may be referred to as *the analytical styles*.

#### Type III intellectual styles

These styles differ considerably from the previous types, since, depending on the demands of a given task and an individual’s personal interest in it, they may produce a degree of preference corresponding to those connected to types I and II mentioned above. Thus, compared to the two other types of style, this type of intellectual styles is loaded with differentiated value and is relatively unstable, i.e. susceptible to influence. With regards to the type III styles, Zhang and Sternberg (2005) give the following example in order to explain the difference between these styles and the previous two types: Depending on the style-related demands of the task and their personal interest in the task, a person with a performance oriented approach to learning will either prefer a low degree of structuring and thereby address the task creatively in a way which resembles type I styles, or the same person could also prefer a high degree of structuring and thereby address the task in a way which resembles type II styles. Collectively, styles placed in this category may be referred to as *the performance and socially oriented styles*.

### Kozhevnikov et al.'s cognitive style matrix

Kozhevnikov et al. (2014) cognitive style matrix also constitutes a type C categorization of style theories, though not a pure type C categorization, as it is a hierarchical categorization of both individual styles across theories and whole theories of styles, resulting in an overall taxonomy of cognitive styles (mixed type B and type C). Kozhevnikov et al.'s cognitive style matrix consists of two axes each representing a set of characteristics, between which a total of 19 theories of styles are placed. The vertical axis represents levels of information processing from the most simple to the most complex (perception, concept formation, higher-order cognitive processing and metacognitive processing). The horizontal axis represents ways of adapting to the external environment (context dependence/independence, rule-based vs. intuitive processing, internal vs. external locus of processing and integration vs. compartmentalization).

The resulting cognitive style matrix consists of 16 fields between the two axis, into which Kozhevnikov et al. place what they term traditional styles, learning styles and decision-making styles (se also discussion). Most theories are placed as the complete theories with all styles (for example reflexivity-impulsivity; Kagan 1966). With other theories, style dimensions are split and placed in different positions in the matrix (for example the dimensions in Gregorc's (1982, 1998) theory of mind styles). Again with other theories, style dimensions are extracted from a theory, for no apparent reason, and placed in the matrix (for example Kolb's (1984) convergent and divergent learning styles).

## Discussion

Zhang and Sternberg’s (2005) categorization was the first successful suggestion for a taxonomy of styles across theories, and as such a first step towards the development of distinct classes of single styles across theories, as Messick (1984) did not succeed equally well in his attempt to categorize styles across theories.

The categorization by Kozhevnikov et al. (2014) represents another attempt to categorize a number of styles across theories, but primarily whole theories, in a unifying model (i.e. the cognitive style matrix) of cognitive styles. Kozhevnikov et al.'s (2014) categorization, however, rather than making the field clearer to practitioners, adds to the disagreements and perhaps contributes further to the confusion of practitioners by defining and labeling style theories in entirely new, and inconsistent, ways. Kozhevnikov et al. raises cognitive style from being but one form of style to instead being an umbrella concept encompassing "traditional cognitive styles", "learning styles" and "decision making styles", somewhat in the same way as Zhang and Sternberg's (2005) proposed the umbrella term "intellectual style". However, as the concept of cognitive styles have always between a distinct category of style theories - even if defined differently by different theorists and researchers - such a change in the use of the concepts is considered confusing rather than elucidating. Also, the single style theories that are placed in the cognitive style matrix are not just "traditional cognitive styles", "learning styles" and "decision making styles", but also include constructs that are not commonly conceived as style constructs, i.e. "intrinsic/extrinsic motivation" in Biggs (1987), which are indeed students motives which in turn affect their learning strategies or styles (Riding and Rayner 2000), and "tolerance for ambiguity" (see Furnham and Ribchester 1995). If fact both of these constructs are categorized first as learning styles and ultimately as cognitive styles in Kozhevnikov et al.'s cognitive styles matrix. Furthermore, Kozhevnikov et al. (2014) appear to define learning styles, as any style constructs (as well as other constructs of individual differences - see above), which has been used to measure styles or individual differences in an educational context. In the same way, Kozhevnikov et al. appear to define decision making styles, as any style construct that has been used to measure styles in a business/management context. This categorization or labeling of style theories exclusively according to the context they are used in, is not only unusual, but indeed in opposition to the main body of work on the nature of the different style theories, where the actual construct and its content has been central. This break with the styles field in general might lead the research field forward, but can only confuse practitioners. It should be mentioned that Koshevnikov et al. do write that the proposed taxonomy is a work in progress to be further developed, and as such it might in the future be developed so that it does provide practitioners with a new "tool" in their search for relavent style theories.

Zhang and Sternberg’s (2005) categorization, is thus found to still be the most comprehensive and useful categorization for practitioners, as it creates new opportunities, both with regards to the exploration of styles and with regards to working with styles in practice. Taking this style taxonomy as one’s point of departure, the practitioner may thus achieve a full overview of the styles in ten theories and how these relate to the six characteristics mentioned, as well as which of the three types of styles they belong to. When used in combination with a stability taxonomy of styles, such as for example Coffield et al.’s (2004) stability taxonomy (cf. Table 2) or Curry's (1981) onion model, this provides practitioners with new possibilities of choice of style theory or single styles, depending on the level of changed which is sought achieved in the specific pedagogical work, as well as of relating the chosen theory to the overall goal one wishes to work towards as a practitioner (with one’s own teaching styles or with one’s students’ learning styles), in relation to the more general terms of style like creative, analytical and performance oriented in Zhang and Sternberg's categorization.

## Closing remarks

The different types of categorizations of theories of styles proposed may provide practitioners with useful information on the overall orientation of theories as well as stability, pedagogical amenability of different styles in connection with the intended use of styles in pedagogical work or other areas of practice. There are, however, subjects within the field of theories of styles equally relevant to practitioners which have not been covered in this paper, due to the extent of these subjects. One such subject is the development which has taken place with regards to measuring instruments for the “uncovering” of styles. Another is the relationship between the different concepts of styles (for example cognitive style and learning style), and the relationship between style and concepts such as abilities and personality. So, even if the present discussion has provided some paths through the “jungle of theories of styles”, additional work concerned with providing practitioners with clear summative knowledge across many more theories are needed in order to make the field of style theories more easily accessible and practically appealing to practitioners. Such future work could utilize the categorizations proposed in this paper, and could focus on subjects of importance to practitioners, such as: overall orientation of theories, stability of styles and pedagogical susceptibility of styles, within different areas of application.

## Authors’ original submitted files for images

Below are the links to the authors’ original submitted files for images. Authors’ original file for figure 1
