# Supplementary figures and images for: Intellectual style theories: different types of categorizations and their relevance for practitioners
Source: Springerplus. 2014 Dec 15;3:737. doi: 10.1186/2193-1801-3-737 (PMC4320160; doi:10.1186/2193-1801-3-737)

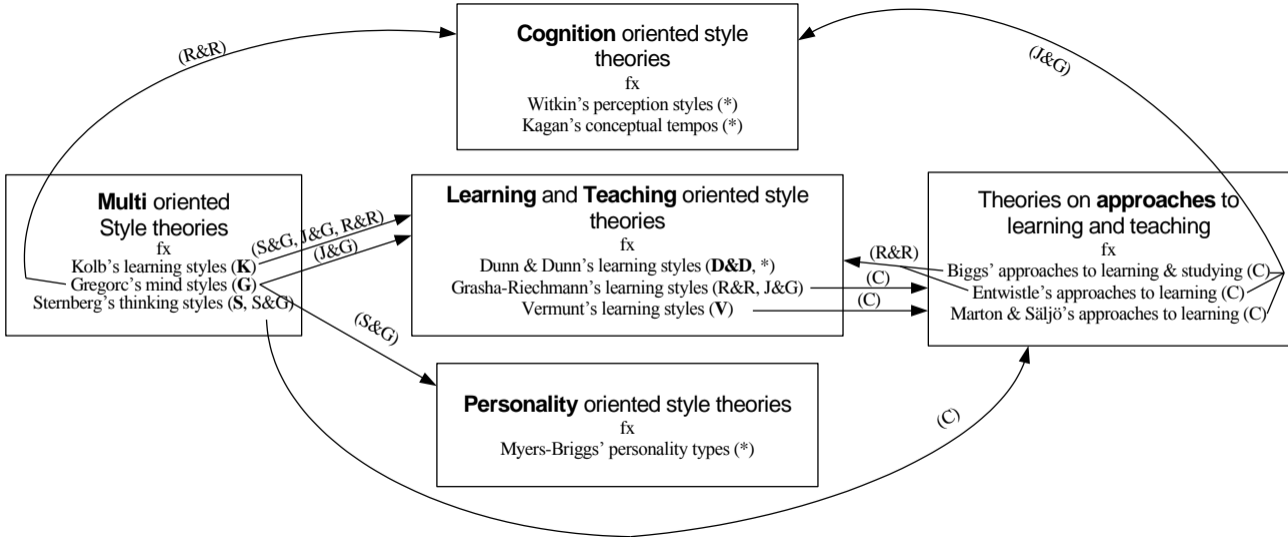

Supplement: Supplementary file 1 — Authors’ original file for figure 1 [file 40064_2013_1499_MOESM1_ESM.pdf]
